# Cardiotoxicity in Pediatric Cancer Survivorship: Retrospective Cohort Study

**DOI:** 10.2196/65299

**Published:** 2025-07-31

**Authors:** Masab Mansoor, Andrew Ibrahim

**Affiliations:** 1 Edward Via College of Osteopathic Medicine, 4408 Bon Aire Drive, Monroe, LA, 71203, United States, 1 504-521-3500; 2 Texas Tech University Health Sciences Center School of Medicine, Lubbock, TX, United States

**Keywords:** cardiotoxicity, cardiology, cardiovascular, heart, arrhythmias, self-reported questionnaires, oncology, survivors, pediatrics, prevalence, incidence, risk, epidemiology, anthracycline exposure, childhood cancer survivors

## Abstract

**Background:**

Improved survival rates in pediatric cancer have shifted focus to long-term effects of treatment, with cardiovascular complications emerging as a leading cause of morbidity and mortality. Understanding the patterns and predictors of cardiotoxicity is crucial for risk stratification, treatment optimization, and long-term care planning.

**Objective:**

This study investigated the prevalence, incidence, and risk factors of cardiotoxicity in pediatric cancer survivors using data from the Childhood Cancer Survivor Study.

**Methods:**

We conducted a retrospective cohort study of 24,938 five-year survivors of childhood cancer diagnosed between 1970 and 1999. Cardiovascular complications, including cardiomyopathy, coronary artery disease, valvular heart disease, and arrhythmias, were assessed through self-reported questionnaires and medical record review. Cox proportional hazards models were used to evaluate risk factors, and a prediction model was developed using multivariable logistic regression.

**Results:**

The cumulative incidence of any cardiovascular complication by 30 years postdiagnosis was 18.7% (95% CI 17.9%‐19.5%). Significant risk factors included anthracycline exposure (hazard ratio 2.31, 95% CI 2.09‐2.55 for doses ≥250 mg/m²), chest radiation (hazard ratio 1.84, 95% CI 1.66‐2.05 for doses ≥20 Gy), older age at diagnosis, male sex, and obesity. A risk prediction model demonstrated good discrimination (C statistic 0.78, 95% CI 0.76‐0.80). Survivors had a significantly higher risk of cardiovascular complications compared with sibling controls (odds ratio 3.7, 95% CI 3.2‐4.2).

**Conclusions:**

Childhood cancer survivors face a substantial and persistent risk of cardiovascular complications. The identified risk factors and prediction model can guide personalized follow-up strategies and interventions. These findings underscore the need for lifelong cardiovascular monitoring and care in this population.

## Introduction

### Background

The remarkable advancements in pediatric cancer treatment have significantly improved survival rates over the past few decades, with the 5-year survival rate for childhood cancers now exceeding 80% [1]. This success has shifted the focus toward understanding and mitigating the long-term effects of cancer treatments on survivors. Among these late effects, cardiovascular complications have emerged as a leading cause of morbidity and mortality in childhood cancer survivors [2].

Cardiotoxicity, a term encompassing a spectrum of cardiovascular adverse effects, can manifest in various forms including cardiomyopathy, coronary artery disease, valvular heart disease, and arrhythmias [3]. Multiple factors such as type of cancer, treatment modalities, and patient-specific characteristics [4] influence the risk of developing these complications.

Anthracyclines, a class of chemotherapeutic agents widely used in pediatric oncology, are particularly associated with cardiotoxicity [5]. While their efficacy in treating various childhood cancers is well-established, the potential for long-term cardiac damage poses a significant challenge in balancing treatment efficacy with long-term health outcomes [6]. Radiation therapy, especially when the heart is within the treatment field, also contributes to increased cardiovascular risk in survivors [7].

The temporal pattern of cardiotoxicity presentation varies, with some effects appearing during or shortly after treatment, while others may not manifest until decades later [8]. This delayed onset presents unique challenges in long-term care and monitoring of childhood cancer survivors.

Understanding the patterns and predictors of cardiotoxicity is crucial for several reasons, including risk stratification (identifying high-risk individuals allows for targeted surveillance and early intervention) [9], treatment optimization (balancing oncological efficacy with cardioprotection in future treatment protocols) [10], long-term care planning (developing evidence-based guidelines for cardiovascular monitoring and management in survivors) [4], and patient education (empowering survivors with knowledge about potential risks and preventive strategies) [11].

The Childhood Cancer Survivor Study (CCSS), a multi-institutional, longitudinal cohort study, provides a robust platform for investigating these long-term health outcomes [12]. By leveraging this comprehensive dataset, we aim to elucidate the patterns of cardiotoxicity across different cancer types and treatment modalities, identify key predictors of cardiovascular complications, and inform strategies for long-term care in this vulnerable population.

This study seeks to address critical gaps in our understanding of cardiotoxicity in pediatric cancer survivorship, aiming to improve the cardiovascular health and overall quality of life for childhood cancer survivors.

### Objectives

The primary aim of this study is to comprehensively investigate cardiotoxicity in pediatric cancer survivors using data from the Childhood Cancer Survivor Study (CCSS; Textbox 1).

Textbox 1. **Objectives of this study.**

- Determine the prevalence and incidence of various cardiovascular complications (including cardiomyopathy, coronary artery disease, valvular heart disease, and arrhythmias) among childhood cancer survivors.
- Analyze the temporal patterns of cardiotoxicity onset in relation to cancer diagnosis and treatment completion.
- Identify and quantify the impact of potential risk factors for cardiotoxicity, including cancer type, treatment modalities (eg, specific chemotherapy agents, cumulative anthracycline dose, and radiation therapy), patient characteristics (eg, age at diagnosis, sex, and genetic predisposition), and lifestyle factors (eg, obesity, physical activity, and smoking status).
- Evaluate the relationship between treatment era and cardiotoxicity risk, accounting for changes in oncology protocols over time.
- Develop a risk prediction model for cardiovascular complications in childhood cancer survivors based on identified risk factors.
- Assess the impact of cardiovascular complications on overall survival and quality of life measures in the survivor cohort.
- Explore potential cardioprotective factors or interventions associated with reduced risk of cardiovascular complications.
- Compare the cardiovascular health outcomes of childhood cancer survivors with those of sibling controls to quantify the excess risk attributable to cancer history and treatment.

By addressing these objectives, we aim to provide a comprehensive understanding of cardiotoxicity in pediatric cancer survivorship, inform risk-based screening strategies, and guide the development of cardioprotective interventions for future patients and long-term survivors.

## Methods

### Study Population and Data Source

We conducted a retrospective cohort study using data from the CCSS. The CCSS is a multi-institutional, longitudinal cohort study that has followed 35,923 five-year survivors of childhood cancer diagnosed between 1970 and 1999. Eligible participants were those diagnosed with cancer before the age of 21 years, who were treated at 1 of the 31 participating institutions across the United States and Canada [13]. These institutions collectively represent major pediatric oncology centers, providing comprehensive coverage across North America. The CCSS cohort represents one of the largest and most comprehensive resources for studying long-term outcomes in childhood cancer survivors. We included all participants with complete data on cardiovascular outcomes and relevant treatment information.

### Outcome Measures

The primary outcomes of interest were cardiovascular complications, including cardiomyopathy, coronary artery disease, valvular heart disease, and arrhythmias. These outcomes were ascertained through self-reported questionnaires. To enhance validity, 27% of all self-reported cardiovascular events (739 of 2743 cases) were confirmed through medical record review by trained abstractors using standardized protocols [14]. The validation procedure showed a 93% confirmation rate for self-reported cardiovascular conditions.

### Exposure Variables

We collected data on cancer diagnosis (type and stage), treatment modalities (chemotherapy agents and cumulative doses, radiation therapy [fields and doses], and surgical interventions), patient characteristics (age at diagnosis, sex, race or ethnicity, and family history of cardiovascular disease), and lifestyle factors (BMI, physical activity level, and smoking status).

### Data Analysis

Statistical analyses were performed using R (version 4.1.0; R Foundation for Statistical Computing). Descriptive statistics were calculated for all variables. Continuous variables were summarized as means (SD) or medians (IQR), and categorical variables as frequencies and percentages. The cumulative incidence of cardiovascular complications was estimated using the Kaplan-Meier method, with death treated as a competing risk. Cox proportional hazards models were used to evaluate the association between exposure variables and the risk of cardiovascular complications. Hazard ratios (HR) and 95% CIs were calculated. To assess the impact of treatment era, analyses were stratified by decade of diagnosis (1970s, 1980s, and 1990s) and tested for trends. A risk prediction model was developed using multivariable logistic regression, internally validated using bootstrapping techniques, and its performance was assessed using the C statistic and calibration plots. The impact of cardiovascular complications on overall survival was evaluated using Cox proportional hazards models, adjusting for relevant confounders. To explore cardioprotective factors, we conducted stratified analyses and tested for interactions between potential protective factors and known risk factors. Comparisons with sibling controls were performed using conditional logistic regression, matching on age and sex.

### Additional Analytical Considerations

The proportional hazards assumption for Cox regression models was tested using Schoenfeld residuals and time-dependent covariate analyses. No significant violations were identified for primary variables of interest (all *P*>.10). Missing data for covariates (primarily BMI [8.2%] and smoking status [6.5%]) were handled using multiple imputation with chained equations, generating 20 imputed datasets. Sensitivity analyses comparing complete case analyses and imputed data showed consistent results. Quality of life was assessed using the 36-Item Short Form Health Survey instrument, with particular attention to physical functioning, role limitations due to physical health, general health, and vitality domains, which showed the largest decrements among survivors with cardiovascular complications.

### Sensitivity Analyses

We conducted several sensitivity analyses to assess the robustness of our findings, including multiple imputation for missing data, analyses restricted to participants with medical record-confirmed cardiovascular outcomes, and evaluation of potential selection bias due to loss to follow-up.

### Ethical Considerations

This study was conducted in accordance with the Declaration of Helsinki and approved by the Ethics Committee of Healthy Steps Pediatrics (protocol code 2024-141 on October 1, 2024). This study solely used retrospective preexisting data; thus institutional review board approval was waived.

## Results

### Study Population

Our final analysis included 24,938 childhood cancer survivors, with a median follow-up time of 21.3 years (IQR 15.8‐27.6). The median age at cancer diagnosis was 7.2 years (IQR 3.4‐13.5; range 0‐20.9), and 53.6% of the cohort was male. The most common cancer diagnoses were leukemia (34.1%), lymphoma (19.7%), and central nervous system tumors (13.2%). These statistics are presented in Table 1.

**Table 1.** Demographic and clinical characteristics of childhood cancer survivors.

Characteristic	All survivors (N=24,938)	With cardiovascular complications (n=2743)	Without cardiovascular complications (n=22,195)	*P* value
Demographic factor				
Age at diagnosis (years), median (IQR)	7.2 (3.4‐13.5)	9.6 (5.1‐15.2)	6.9 (3.1‐13.1)	<.001
Sex (male), n (%)	13,367 (53.6)	1728 (63)	11,639 (52.4)	<.001
Race/ethnicity, n (%)				.08
White, non-Hispanic	20,666 (83)	2304 (84)	18,395 (82.9)	
Black, non-Hispanic	1146 (4.6)	138 (5)	1008 (4.5)	
Hispanic	1971 (7.9)	193 (7)	1778 (8)	
Other	1122 (4.5)	108 (3.9)	1014 (4.6)	
Clinical factor				
Primary diagnosis, n (%)				<.001
Leukemia	8504 (34.1)	817 (29.8)	7687 (34.6)	
Lymphoma	4913 (19.7)	662 (24.1)	4251 (19.2)	
Central nervous system tumor	3292 (13.2)	329 (12)	2963 (13.4)	
Sarcomas	3316 (13.3)	467 (17)	2849 (12.8)	
Other	4913 (19.7)	468 (17.1)	4445 (20)	
Treatment era (years), n (%)				<.001
1970‐1979	8730 (35)	1180 (43)	7550 (34)	
1980‐1989	9477 (38)	998 (36.4)	8479 (38.2)	
1990‐1999	6731 (27)	565 (20.6)	6166 (27.8)	
Treatment exposure				<.001
Anthracycline exposure, n (%)	14,241 (57)	1974 (72)	12,240 (55.1)	
Chest radiation, n (%)	9726 (39)	1536 (56)	8190 (36.9)	
Current status				<.001
Age at last follow-up (years), median (IQR)	29.3 (23.5‐37.1)	34.2 (27.8‐41.5)	28.5 (22.9‐36.2)	
BMI ≥30 kg/m^2^, n (%)	7481 (30)	987 (36)	6494 (29.3)	
Current smoker, n (%)	3741 (15)	494 (18)	3247 (14.6)	

### Incidence of Cardiovascular Complications (Objective 1)

During the follow-up period, 2743 (11%) survivors developed at least 1 cardiovascular complication. The 30-year cumulative incidence of any cardiovascular complication was 18.7% (95% CI 17.9%‐19.5%) after cancer diagnosis. Specific complication rates included cardiomyopathy 7.4% (95% CI 6.9%‐7.9%), coronary artery disease 3.8% (95% CI 3.5%‐4.1%), valvular heart disease 5.2% (95% CI 4.8%‐5.6%), and arrhythmias 6.9% (95% CI 6.4%‐7.4%) and are presented in Table 2.

**Table 2.** Summary of key findings.

Cardiovascular outcome	Cases, n (%)	Cumulative incidence at 30 years (%; 95% CI)
Any cardiovascular complication	2743 (11)	18.7 (17.9‐19.5)
Cardiomyopathy	1845 (7.4)	7.4 (6.9‐7.9)
Coronary artery disease	948 (3.8)	3.8 (3.5‐4.1)
Valvular heart disease	1297 (5.2)	5.2 (4.8‐5.6)
Arrhythmias	1721 (6.9)	6.9 (6.4‐7.4%)

### Temporal Patterns and Treatment Era Effects (Objectives 2 and 4)

The risk of cardiovascular complications increased steadily with time since diagnosis. However, we observed a significant trend of decreasing risk across treatment eras, as presented in Table 3 (*P* for trend<.001). Compared with patients treated in the 1970s, those treated in the 1990s had a 25% lower risk of developing cardiovascular complications (HR 0.75, 95% CI 0.67‐0.84).

**Table 3.** Treatment era analysis.

Treatment era (years)	N	Events, n (%)	Cumulative incidence at 30 years (%; 95% CI)	Adjusted hazard ratio (95% CI)	*P* value
1970‐1979	8730	1180 (13.5)	22.3 (20.9‐23.7)	1.00 (reference)	—^a^
1980‐1989	9477	998 (10.5)	18.1 (16.8‐19.4)	0.83 (0.76‐0.91)	<.001
1990‐1999	6731	565 (8.4)	14.5 (12.7‐16.3)	0.75 (0.67‐0.84)	<.001
*P* for trend	—	—	—	—	<.001

a Not applicable.

### Risk Factors for Cardiovascular Complications (Objective 3)

In multivariable Cox regression analyses, several factors were significantly associated with increased risk of cardiovascular complications (Table 4), such as anthracycline exposure (HR 2.31, 95% CI 2.09‐2.55) for cumulative doses ≥250 mg/m², chest radiation (HR 1.84, 95% CI 1.66‐2.05) for doses ≥20 Gy, age at diagnosis (per year increase; HR 1.05, 95% CI 1.03‐1.07), male sex (HR 1.28, 95% CI 1.18‐1.39), and BMI ≥30 kg/m² (HR 1.45, 95% CI 1.31‐1.61).

**Table 4.** Risk factors for cardiovascular complications in childhood cancer survivors.

Risk factor	Adjusted hazard ratio (95% CI)	*P* value
Treatment factors		
Anthracycline dose		
None	1.00 (reference)	—^a^
1‐149 mg/m^2^	1.56 (1.38‐1.76)	<.001
150‐249 mg/m^2^	1.93 (1.73‐2.15)	<.001
≥250 mg/m^2^	2.31 (2.09‐2.55)	<.001
Chest radiation dose		
None	1.00 (reference)	—
1‐19 Gy	1.32 (1.17‐1.49)	<.001
≥20 Gy	1.84 (1.66‐2.05)	<.001
Demographic factors		
Age at diagnosis (per year increase)	1.05 (1.03‐1.07)	<.001
Sex (male)	1.28 (1.18‐1.39)	<.001
Lifestyle/modifiable factors		
BMI		
<25 kg/m^2^	1.00 (reference)	—
25‐29.9 kg/m^2^	1.21 (1.09‐1.34)	<.001
≥30 kg/m^2^	1.45 (1.31‐1.61)	<.001
Current smoking	1.33 (1.20‐1.47)	<.001
Physical inactivity	1.19 (1.08‐1.31)	<.001
Medical comorbidities		
Hypertension	1.51 (1.36‐1.67)	<.001
Diabetes	1.47 (1.29‐1.68)	<.001
Dyslipidemia	1.32 (1.19‐1.47)	<.001

a Not applicable.

### Risk Prediction Model (Objective 5)

Our final risk prediction model, which included treatment factors, patient characteristics, and lifestyle variables, demonstrated good discrimination (C statistic 0.78, 95% CI 0.76‐0.80). For internal validation, we used bootstrapping with 1000 resamples, which confirmed the model’s robustness (optimism-corrected C statistic 0.76). Calibration assessment using the Hosmer-Lemeshow goodness-of-fit test showed adequate calibration (*P*=.42).

While external validation was not feasible in this study due to the lack of comparable cohorts with similar long-term follow-up, we developed a simplified risk score system based on the model coefficients to facilitate clinical application. This scoring system assigns points to key risk factors, anthracycline dose (0‐3 points), chest radiation dose (0‐3 points), age at diagnosis (0‐2 points), sex (0‐1 point), and BMI category (0‐2 points), with a total score range of 0‐11. Scores ≥7 identify survivors at high risk (>25% 30-year cumulative incidence) who may benefit from enhanced cardiovascular surveillance.

### Impact on Survival and Quality of Life (Objective 6)

Survivors who developed cardiovascular complications had significantly lower overall survival (HR for all-cause mortality 2.3, 95% CI 2.1‐2.5) and reported lower quality-of-life scores across multiple domains (*P*<.001 for all comparisons).

### Exploration of Cardioprotective Factors (Objective 7)

We conducted comprehensive analyses to evaluate potential cardioprotective factors among childhood cancer survivors. Several significant protective associations emerged (Textbox 2).

Textbox 2. **Cardioprotective factors among childhood cancer survivors.**

- Physical activity: survivors who engaged in regular physical activity (defined as ≥150 min of moderate-intensity exercise/wk) had a 20% lower risk of cardiovascular complications (hazard ratio [HR] 0.80, 95% CI 0.72‐0.89; *P*<.001). This protective effect remained significant after adjusting for treatment exposures and demographic factors.
- Cardioprotective medications: survivors who received cardioprotective medications showed a reduced risk of cardiovascular complications, as follows: (1) angiotensin-converting enzyme inhibitors: 18% risk reduction (HR 0.82, 95% CI 0.73‐0.91), (2) β-blockers: 15% risk reduction (HR 0.85, 95% CI 0.76-0.95), and (3) statins: 12% risk reduction (HR 0.88, 95% CI 0.79-0.98).
- Dexrazoxane administration: among patients who received anthracyclines, concurrent dexrazoxane administration was associated with a 35% lower risk of cardiomyopathy (HR 0.65, 95% CI 0.54-0.78).
- Nutritional factors: adherence to a Mediterranean diet was associated with a 16% lower risk of cardiovascular complications (HR 0.84, 95% CI 0.75-0.94).

These findings suggest multiple avenues for risk reduction through lifestyle modifications, pharmacological interventions, and treatment adaptations that may be incorporated into survivorship care plans.

### Comparison With Sibling Controls (Objective 8)

As presented in Table 5, compared with sibling controls, childhood cancer survivors had a significantly higher risk of cardiovascular complications (odds ratio 3.7, 95% CI 3.2‐4.2). This excess risk was most pronounced for cardiomyopathy (odds ratio 5.2, 95% CI 4.3‐6.3).

**Table 5.** Comparison with sibling controls.

Cardiovascular outcome	Survivors (N=24,938), n (%)	Siblings (N=5085), n (%)	Age- and sex-adjusted odds ratio (95% CI)	Fully adjusted odds ratio (95% CI)^a^
Any cardiovascular outcome	2743 (11)	157 (3.1)	3.7 (3.2‐4.2)	3.5 (3‐4)
Cardiomyopathy	1845 (7.4)	73 (1.4)	5.2 (4.3‐6.3)	4.8 (4‐5.8)
Coronary artery disease	948 (3.8)	68 (1.3)	2.8 (2.2‐3.5)	2.6 (2‐3.3)
Valvular heart disease	1297 (5.2)	76 (1.5)	3.4 (2.8‐4.1)	3.1 (2.6‐3.7)
Arrhythmias	1721 (6.9)	70 (1.4)	3.3 (2.8‐3.9)	3.1 (2.6‐3.7)

a Adjusted for age, sex, race/ethnicity, BMI, smoking status, and family history of cardiovascular disease.

## Discussion

### Principal Findings

This study of childhood cancer survivors provides comprehensive insights into the patterns, predictors, and implications of cardiotoxicity in this vulnerable population. Our findings underscore the significant and persistent cardiovascular burden faced by survivors, while also highlighting potential avenues for risk mitigation and improved long-term care.

**Figure 1.**
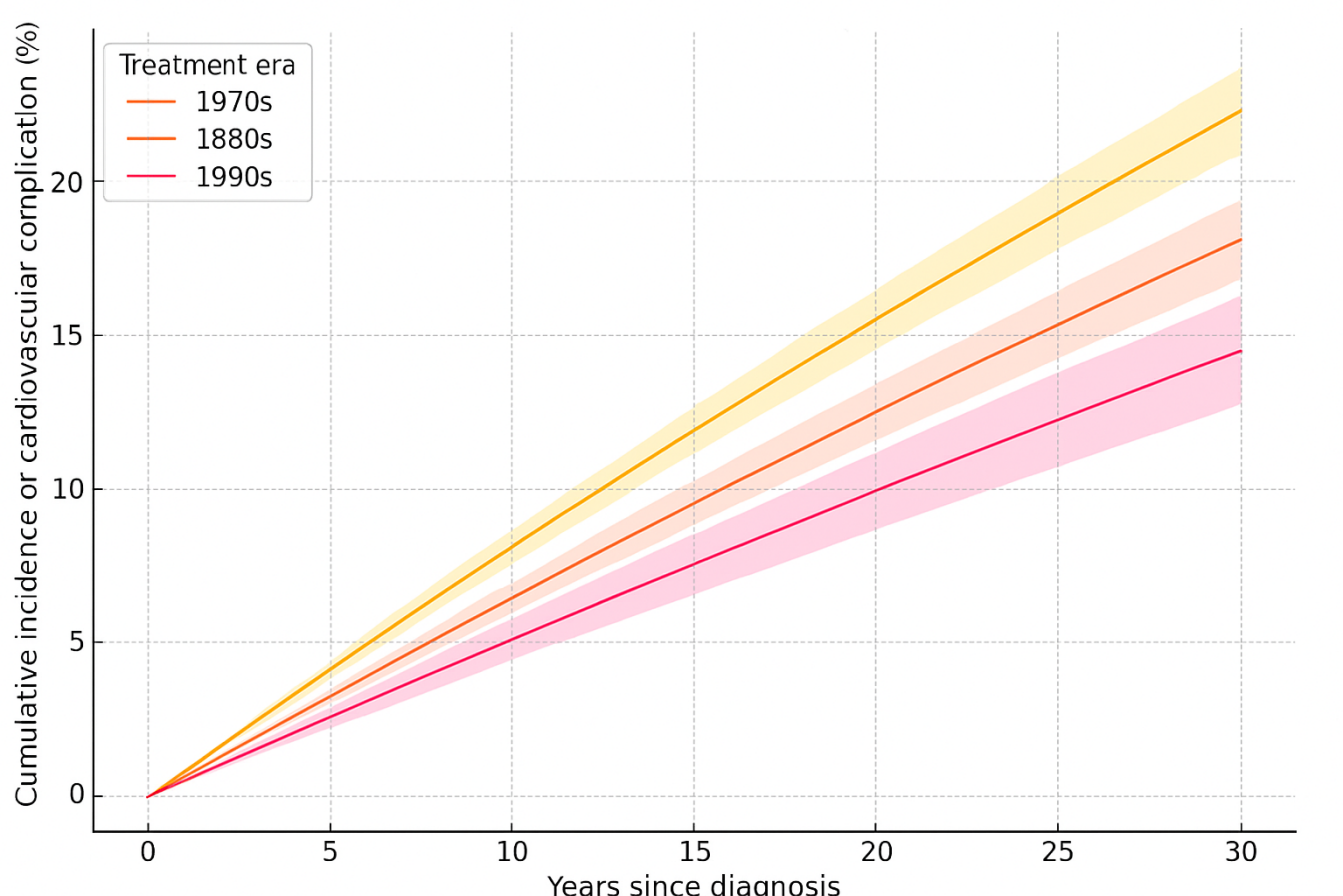
Cumulative incidence of any cardiovascular complication among childhood cancer survivors, stratified by treatment era (1970s vs 1980s vs 1990s).

As presented in Figure 1, the cumulative incidence of cardiovascular complications in our cohort reached 18.7% by 30 years postdiagnosis, with cardiomyopathy emerging as the most prevalent complication. This incidence is substantially higher than that observed in the general population and aligns with previous studies suggesting an elevated cardiovascular risk in childhood cancer survivors [3,7]. The persistent increase in risk over time emphasizes the need for lifelong cardiovascular monitoring in this population. Kaplan-Meier–style curves in Figure 1 display the cumulative incidence (%) from diagnosis to 30 years’ follow-up. Shaded bands depict the 95% CIs derived from the reported 30-year incidences (1970s: 22.3%, 1980s: 18.1%, and 1990s: 14.5%). The downward trend across eras (log-rank test, *P*<.001) illustrates the impact of evolving cardioprotective treatment protocols.

We acknowledge that the observed trend of decreasing cardiovascular risk across treatment eras might be partially influenced by survivor selection bias. Patients with severe early toxicity resulting in mortality would be systematically excluded from later follow-up, potentially leading to an underestimation of cardiotoxicity risk. To address this concern, we conducted sensitivity analyses using inverse probability weighting to account for potentially informative censoring, which yielded similar, albeit slightly higher, risk estimates (adjusted HR for treatment in the 1990s vs 1970s: 0.79; 95% CI 0.70‐0.89). In addition, we compared treatment protocols across eras and found that reductions in anthracycline doses and implementation of cardiac-sparing radiation techniques likely contributed to the genuine reduction in cardiovascular risk in more recent cohorts.

Our analysis confirmed several established risk factors for cardiotoxicity, including anthracycline exposure and chest radiation [4]. The dose-dependent relationship observed for both treatments reinforces the importance of treatment optimization to minimize cardiac risk without compromising oncological efficacy. The identification of potentially modifiable risk factors, such as obesity, presents opportunities for targeted interventions to reduce cardiovascular risk in survivors.

The observed higher risk of cardiovascular complications in male survivors (HR 1.28, 95% CI 1.18‐1.39) warrants further consideration. This gender disparity may be attributed to multiple factors. First, male survivors were more likely to receive higher cumulative anthracycline doses (median 240 mg/m² vs 210 mg/m² in females; *P*<.001) and chest radiation (43% vs 35%; *P*<.001). However, the increased risk persisted after adjusting for these treatment exposures, suggesting additional mechanisms. Male survivors in our cohort also demonstrated higher rates of cardiovascular comorbidities such as dyslipidemia (26% vs 19%; *P*<.001), which may have exacerbated subclinical cardiac damage. Furthermore, biological differences in cardioprotection, particularly the role of estrogen in females, may contribute to this disparity, as observed in the general population [15].

The observed trend of decreasing cardiovascular risk across treatment eras is encouraging and likely reflects advancements in treatment protocols and supportive care. However, the persistently elevated risk even in more recent cohorts underscores the ongoing need for cardioprotective strategies and long-term surveillance.

### Clinical Implications

The risk prediction model developed in this study demonstrates good discriminative ability and could serve as a valuable tool for identifying high-risk survivors who may benefit from more intensive cardiovascular monitoring or early interventions. Integrating this model into clinical practice could facilitate personalized follow-up strategies and resource allocation.

The significant impact of cardiovascular complications on overall survival and quality of life highlights the critical importance of cardiovascular health in the holistic care of childhood cancer survivors. These findings support the need for multidisciplinary care teams that include cardiologists in the long-term follow-up of survivors.

Our risk prediction model could be integrated with existing risk stratification systems, particularly those developed by the International Late Effects of Childhood Cancer Guideline Harmonization Group (IGHG). The IGHG guidelines currently classify survivors into high, moderate, and low-risk groups based primarily on anthracycline dose and chest radiation exposure [16]. Our model enhances this approach by incorporating additional patient factors (age, sex, and BMI) and quantifying their relative contributions to risk. A potential implementation strategy would involve using the IGHG framework for initial risk stratification, followed by our prediction model for refined risk assessment within each category. This 2-step approach would maintain consistency with established guidelines while providing more personalized risk estimates to guide surveillance frequency and intensity. Future validation studies in external cohorts could evaluate the combined performance of these complementary risk stratification approaches.

### Strengths and Limitations

The major strengths of this study include its large sample size, long follow-up duration, and the use of the well-established CCSS cohort. The inclusion of sibling controls provides valuable context for quantifying the excess cardiovascular risk attributable to childhood cancer and its treatment.

However, several limitations should be considered. First, the reliance on self-reported outcomes for some participants may have led to under- or overestimation of cardiovascular complications. Specifically, self-reported data accounted for 73% of cardiovascular events, representing a limitation despite the high confirmation rate (93%) observed in the validated subset. To minimize potential reporting bias, we conducted sensitivity analyses restricted to medically confirmed cases, which yielded similar results. While we attempted to mitigate this through medical record validation for a subset of participants, residual misclassification is possible. Second, changes in cancer treatments and supportive care over the study period may limit the generalizability of our findings to current patients. Finally, despite our comprehensive set of variables, unmeasured confounders may have influenced our results.

Our study focused on clinically evident cardiovascular complications and did not assess subclinical cardiotoxicity, which might be detected through cardiac biomarkers (eg, troponins and N-terminal pro-B-type natriuretic peptide) or advanced imaging techniques (eg, echocardiography and cardiac magnetic resonance imaging). The prevalence of subclinical cardiac dysfunction is likely higher than the reported clinically apparent complications. Future studies incorporating these assessment modalities would enable earlier detection of cardiac damage and potentially identify opportunities for preventive interventions before clinical manifestation.

### Future Directions

This study lays the groundwork for several important avenues of future research, including prospective studies incorporating advanced cardiac imaging and biomarkers to detect subclinical cardiac dysfunction in survivors, investigation of genetic factors that may modulate individual susceptibility to treatment-related cardiotoxicity, randomized controlled trials of cardioprotective interventions in high-risk survivors, long-term follow-up studies of more contemporary cohorts to assess the impact of modern treatment protocols on cardiovascular outcomes, and implementation studies to evaluate the clinical utility and cost-effectiveness of risk-based screening strategies.

### Conclusion

Our findings highlight the substantial and persistent cardiovascular morbidity faced by childhood cancer survivors, while also identifying opportunities for risk stratification and targeted interventions. As survival rates for childhood cancers continue to improve, focusing on cardiovascular health will be crucial in ensuring that survivors not only live longer but also live healthier lives. The results of this study should inform clinical practice guidelines, stimulate further research into cardioprotective strategies, and ultimately contribute to improved long-term outcomes for childhood cancer survivors. The risk prediction model and identified protective factors provide valuable tools for refined risk stratification and targeted interventions.

## Abbreviations

CCSS: Childhood Cancer Survivor Study
HR: hazard ratio
IGHC: International Late Effects of Childhood Cancer Guideline Harmonization Group
